# Polyclonal Dissemination of OXA-232 Carbapenemase–Producing *Klebsiella pneumoniae*, France, 2013–2021

**DOI:** 10.3201/eid2811.221040

**Published:** 2022-11

**Authors:** Cecile Emeraud, Aurélien Birer, Delphine Girlich, Agnès B. Jousset, Elodie Creton, Thierry Naas, Rémy A. Bonnin, Laurent Dortet

**Affiliations:** Institut National de la Santé et de la Recherche Médicale, University Paris-Saclay, Le Kremlin-Bicêtre, France (C. Emeraud, D. Girlich, A.B. Joussett, T. Naas, R.A. Bonnin, L. Dortet); Microbes, Intestin, Inflammation et Susceptibilité de l'Hôte, Clermont–Ferrand, France (A. Birer);; Gabriel–Montpied Hospital, Clermont–Ferrand, France (A. Birer);; Bicêtre Hospital, Le Kremlin-Bicêtre (C. Emeraud, A.B. Jousset, T. Naas, L. Dortet);; National Reference Center for Antibiotic Resistance, Le Kremlin-Bicêtre (C. Emeraud, A. Birer, A.B. Jousset, E. Creton, T. Naas, R.A. Bonnin, L. Dortet);; Gabriel–Montpied Hospital, Clermont–Ferrand, France (A. Birer)

**Keywords:** carbapenemase, Enterobacterales, oxacillin, OXA-232, *Klebsiella pneumoniae*, epidemiology, France, antimicrobial resistance

## Abstract

During 2013–2021, increased prevalence of oxacillinase 232–producing Enterobacterales was observed in France, mostly driven by its emergence in *Klebsiella pneumoniae*. Whole-genome sequencing identified that oxacillinase 232–producing *K. pneumoniae* belonged to 14 sequence types (STs), among which 2 polyclonal high-risk clones, ST-231 and ST-2096, were overrepresented.

The massive dissemination of carbapenemase-producing Enterobacterales poses a global threat to public health. Carbapenem antibiotics remain the last line of defense against highly resistant Enterobacterales. Carbapenemases have been identified in 3 of the 4 classes of the Ambler classification: class A carbapenemases (mostly *Klebsiella pneumoniae* carbapenemase types) (*1*), class B carbapenemases or metallo-β-lactamases (mostly New Delhi metallo-β-lactamase [NDM], Verona integron-mediated metallo-β-lactamase [VIM], or imipenemase types) (*2*), and class D carbapenemases (mostly oxacillinases [OXAs] of OXA-48 types) (*3*). In France, the most prevalent carbapenemases are of OXA-48 type (*4*). According to the Beta-Lactamase Database (http://www.bldb.eu), >50 OXA-48–like carbapenemase variants have been identified. OXA-48, OXA-162, OXA-181, OXA-232, OXA-204, and OXA-244 are the most common enzymes identified among these carbapenemases (*4*).

OXA-232 differs from OXA-181 by a single amino acid substitution (Arg214Ser), differing itself from OXA-48 by 4 substitutions (Thr104Ala, Asn110Asp, Glu168Gln, and Ser171Ala). OXA-232 has been demonstrated to possess a weaker hydrolytic activity toward carbapenems but a stronger ability to hydrolyze penicillins compared with OXA-48 and OXA-181 (*5*,*6*). The *bla*_OXA-232_ gene usually is located on a 6-kb nonconjugative ColE-type plasmid within a truncated Tn*2013*-like transposon (*5*). Furthermore, the genetic environment surrounding the *bla*_OXA-232_ gene is comparable to that of the *bla*_OXA-181_ gene, suggesting that OXA-232 is derived directly from OXA-181 (*4*).

Previous research has mainly identified OXA-232 in *Escherichia coli* and *K. pneumoniae* isolates and has found that this variant is endemic in China, India, South Korea, and Thailand (*4*,*7*,*8*). For *K. pneumoniae,* several outbreaks have been reported with different sequence types (STs), including ST-14, ST-15, ST-16, ST-23, ST-231, and ST-437 (*4*,*9*–*11*). Moreover, to the best of our knowledge, there are no data from France regarding OXA-232 outbreaks and epidemiology since the first description of 1 *E. coli* ST-2968 and 2 *K. pneumoniae* ST-14 isolates from patients returning to France from India in 2012 (*5*).

In addition, strains coproducing NDM and OXA-232 have been reported in several countries (*12*–*14*). In these strains, *bla*_NDM_ and *bla*_OXA-232_ are carried by 2 different plasmids (*13*). The *bla*_OXA-232_ gene is located on a ColE-type plasmid, whereas the *bla*_NDM_ gene usually is carried by an incF-type plasmid (*8*).

Given the increasing prevalence of OXA-232–producing Enterobacterales in Europe, it is crucial to better understand the driving forces of such dissemination. In this study, we used whole-genome sequencing to decipher the epidemiology of OXA-232–producing *K. pneumoniae* in France during 2013–2021.

## The Study

**Figure 1:**
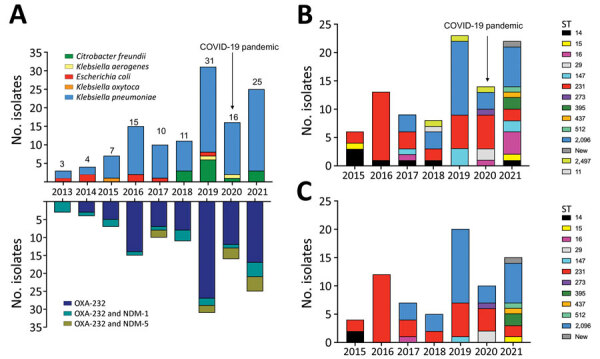
OXA-232–producing Enterobacterales received at the National Reference Center for Carbapenem-Resistant Enterobacterales, France 2013–2021. A) Evolution of several OXA-232–producing Enterobacterales, by species (top of panel) and carbapenemase variant (bottom). B) Evolution of distribution of ST among all OXA-232–producing *K. pneumoniae*. C) Evolution of distribution of ST among NDM and OXA-232–coproducing *K. pneumoniae.* NDM, New Delhi metallo-β-lactamase; OXA, oxacillinase; ST, sequence type.

During 2013–2021, France’s National Reference Centre received 122 nonduplicate OXA-232–producing Enterobacterales, including 99 *K. pneumoniae*, 13 *Citrobacter freundii*, 7 *E. coli*, 2 *K. aerogenes,* and 1 *K. oxytoca* (Figure 1, panel A; Appendix Table 1). These clinical isolates were cultured from rectal swabs (n = 92), urine samples (n = 18), blood cultures (n = 2), respiratory tracts samples (n = 1), and other or unknown origins (n = 9) (Appendix Table 1).

Among these strains, 16 coproduced NDM-1 and 9 coproduced NDM-5 (Figure 1, panel A). Overall, the prevalence of OXA-232 among OXA-48–like producers was significantly higher during 2019–2021 (1.33% among OXA-48–like) compared to 2013–2018 (0.70% among OXA-48–like) (χ^2^ test, p<0.05) (Figure 1, panel A; Appendix Table 2). The prevalence of NDM and OXA-232–coproducing isolates also slightly increased (0.15% among NDM and 0.27% among OXA-48–like from 2013–2018 to 2019–2021) (Figure 1, panel A; Appendix Table 2).

We performed short-read next-generation sequencing on all *K. pneumoniae* strains producing OXA-232 during 2015–2021 (n = 95) using a HiSeq system (Illumina, https://www.illumina.com) and submitted them to GenBank (Appendix Table 1). We assembled Illumina reads using shovill 1.1.0 (https://github.com/tseemann/shovill) and SPAdes 3.14.0 (http://bioinf.spbau.ru/spades) multilocus sequence typing programs, and we performed resistome analysis using pubMLST (https://pubmlst.org) and Resfinder (https://cge.cbs.dtu.dk/services/ResFinder). For phylogenetic analysis, we mapped next-generation sequencing reads to the reference genome (K. pneumoniae HS11286 [GenBank accession no. NC_016845.1]) using SNIppy 4.6.0 (https://software.cqls.oregonstate.edu/updates/snippy-4.6.0). We visualized metadata and phylogenetic trees using iTOL 6.5.2 (https://itol.embl.de).

**Figure 2:**
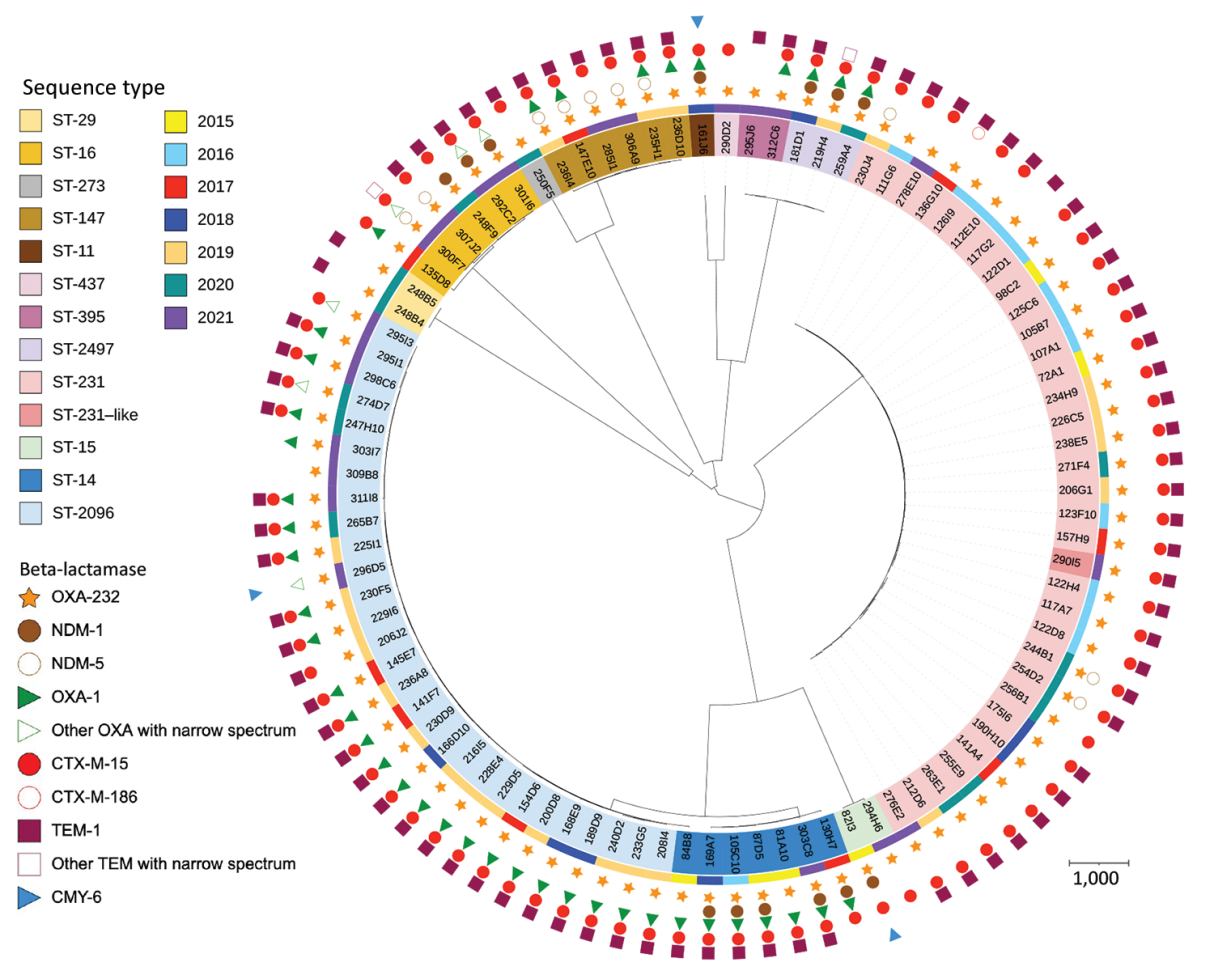
Phylogenetic relationship of OXA-232–producing *K. pneumoniae* ST-231 (A) and ST-2096 (B) analyzed at the National Reference Center for Carbapenem-Resistant Enterobacterales, France 2013–2021.The phylogenetic trees were built with an SNP analysis approach. Scale bars under trees indicate the number of SNPs per position of common sequences. OXA, oxacillinase; SNP, single nucleotide polymorphism; ST, sequence type.

Among the 95 patients colonized or infected with OXA-232–producing *K. pneumoniae*, 19 had recently returned from Asia (including 15 from India) and 12 from the Middle East. Among *K. pneumoniae* isolates, we identified 14 different STs, 5 of which were represented by >5 strains: ST-231 (n = 33), ST-2096 (n = 29), ST-14 (n = 7), ST-16 (n = 6), and ST-147 (n *=* 6). We observed a diversification in OXA-232–producing *K. pneumoniae* STs over the last 2 years of the study period. In addition, the number of *K. pneumoniae* ST-231 isolates decreased, whereas the number of *K. pneumoniae* ST-2096 isolates increased (Figure 1, panel B). We built single nucleotide polymorphism (SNP) matrices and phylogenetic trees for the 2 main STs (ST-231 and ST-2096) and compared them to epidemiologic data. We considered 2 isolates to be clonally related (probably by cross-transmission) if they differed by <21 SNPs, as previously reported for *K. pneumoniae* clonal complex 258 (*15*). For both STs, we identified many subclones (20 for ST-231 and 21 for ST-2096) (Figure 2), suggesting polyclonal dissemination including within these 2 high-risk clones.

**Figure 3:**
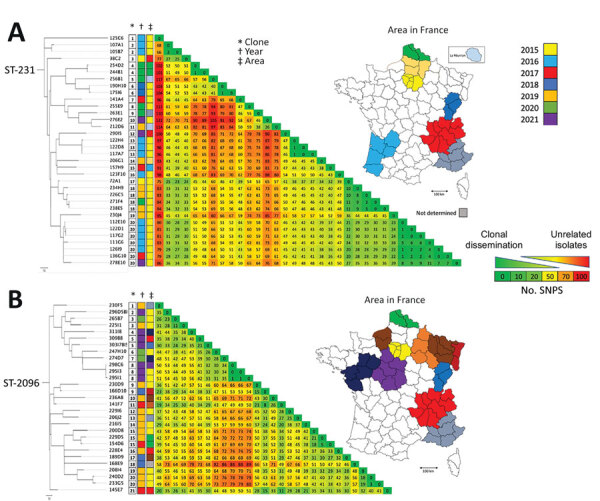
Global characterization (sequence type, year of isolation, β-lactamase content) of nonduplicate 95 OXA-232–producing *Klebsiella pneumoniae* analyzed at the National Reference Center for Carbapenem-Resistant Enterobacterales, France, 2013–2021. Scale bar indicates the number of SNP per position of common sequences. CMY-6, variant of *C. freundii* intrinsic cephalosporinase; CTX-M, cefotaximase–Munich extended-spectrum β-lactamase; OXA, oxacillinase; NDM, New Delhi metallo-β-lactamase; ST, sequence type, TEM, Temoniera β-lactamase.

*K. pneumoniae* coproducing OXA-232 and NDM (NDM-1 or NDM-5) belonged to several STs (ST-14, ST-16, ST-147, ST-231, and ST-2497) but not to ST-2096 (Figure 1, panel C; Appendix Figure 2, 3). Among the 95 OXA-232–producing *K. pneumoniae*, we identified additional β-lactamases in all strains except 1 (309B8). Eighty-two coproduced Temoniera β-lactamase 1 (32/33 for ST-231 and 25/29 for ST-2096), 86 coproduced the cefotaximase–Munich extended-spectrum β-lactamase 15 (31/33 for ST-231 and 26/29 for ST-2096), and 42 coproduced OXA-1 (0/33 for ST-231 and 25/29 for ST-2096) (Figure 3). Furthermore, 3 non–clonally related isolates coproduced the acquired *C. freundii* intrinsic cephalosporinase 6 (ST-231, ST-11, and ST-15) (Figure 3). Analysis of the genetic environment revealed that the *bla*_OXA-232_ was carried by the 6-kb in size ColE-type plasmid as previously described (*5*).

## Conclusions

Recent data suggested that the dissemination of OXA-232–producing *K. pneumoniae* is increasing rapidly, especially in Asia and the Middle East (*7*,*11*). In our study, about a third of patients had recently visited 1 of these regions. Furthermore, we observed an increasing number of OXA-232 and NDM coproducers. These isolates are of high concern because of their lack of susceptibility to all antimicrobials, including last-resort combinations such as ceftazidime/avibactam, meropenem/vaborbactam, and imipenem/relebactam.

The OXA-232–producing *K. pneumoniae* isolates that are reported to be responsible for outbreaks usually belonged to ST-231, ST-15, ST-16 and ST-147 (*4*,*9*). In our study, a wide diversity of STs was found, but the 2 main types were ST-231 and ST-2096. ST-231 was widely reported with OXA-232–producing *K. pneumoniae*, but ST-2096 was first reported only recently in India in 2019 (*7*,*9*). ST-2096 in India was also reported to be hypervirulent because it produced characteristic virulence genes such as *rmpA2*, *iutA*, and *iuc* operon (*9*). Our results suggest that the ST-2096 appeared very recently in France (2017). SNPs analysis demonstrated that the emergence and rapid dissemination of ST-2096 OXA-232–producing *K. pneumoniae* is not linked to a single or a few outbreaks. In our collection, 29 of the 30 ST-2096 *K. pneumoniae* isolates produced OXA-232, whereas the remaining isolate did not produce any carbapenemase, suggesting a recent acquisition of *bla*_OXA-232_ in this clone.

A recent publication reported an association between ST-2096 and a higher risk for bacteriemia and death (*7*). In our study, the unique isolate responsible for bacteriemia belonged to ST-231. In contrast, 25 of the 29 ST-2096 isolates were cultured from rectal swabs.

As expected, *bla*_OXA-232_ was located on a ColE plasmid in all isolates. The close genetic environment of *bla*_OXA-232_ involved IS*Ecp1* upstream of the *bla*_OXA-232_ gene as previously described (*5*).

Appendix Additional information about polyclonal dissemination of OXA-232 carbapenemase–producing *Klebsiella pneumoniae*, France, 2013–2021.
