# Supplementary material for: Polyclonal Dissemination of OXA-232 Carbapenemase–Producing Klebsiella pneumoniae, France, 2013–2021
Source: Emerg Infect Dis. 2022 Nov;28(11):2304–7. doi: 10.3201/eid2811.221040 (PMC9622251; doi:10.3201/eid2811.221040)
Supplement: Appendix — Additional information about polyclonal dissemination of OXA-232 carbapenemase–producing Klebsiella pneumoniae, France, 2013–2021. [file 22-1040-Techapp-s1.pdf]

Article DOI: https://doi.org/10.3201/eid2811.221040

Polyclonal Dissemination of OXA-232 Carbapenemase–Producing *Klebsiella pneumoniae*, France, 2013–2021

Appendix

Appendix Table 1. OXA-232-producing isolates included in the study

Strain	Species	Year	Origin	Carbapenemase	ST	Genome Accession Number
72A1	*K. pneumoniae*	2015	Rectal swab	OXA-232	231	JALXFX000000000
81A10	*K. pneumoniae*	2015	Rectal swab	OXA-232	14	JALXFY000000000
82I3	*K. pneumoniae*	2015	Rectal swab	OXA-232 + NDM-1	15	JALXFZ000000000
84B8	*K. pneumoniae*	2015	Rectal swab	OXA-232	14	JALXGA000000000
87D5	*K. pneumoniae*	2015	Rectal swab	OXA-232 + NDM-1	14	JALXGB000000000
98C2	*K. pneumoniae*	2015	Rectal swab	OXA-232	231	JALXFW000000000
105B7	*K. pneumoniae*	2016	Blood culture	OXA-232	231	JALXGC000000000
105C10	*K. pneumoniae*	2016	Rectal swab	OXA-232 + NDM-1	14	JALXGD000000000
107A1	*K. pneumoniae*	2016	Rectal swab	OXA-232	231	JALXGE000000000
111G6	*K. pneumoniae*	2016	Other or unknown origin	OXA-232	231	JALXGF000000000
112E10	*K. pneumoniae*	2016	Rectal swab	OXA-232	231	JALXGG000000000
117A7	*K. pneumoniae*	2016	Respiratory tract	OXA-232	231	JALXGH000000000
117G2	*K. pneumoniae*	2016	Rectal swab	OXA-232	231	JALXGI000000000
122D1	*K. pneumoniae*	2016	Rectal swab	OXA-232	231	JALXGJ000000000
122D8	*K. pneumoniae*	2016	Rectal swab	OXA-232	231	JALXGK000000000
122H4	*K. pneumoniae*	2016	Rectal swab	OXA-232	231	JALXGL000000000
123F10	*K. pneumoniae*	2016	Rectal swab	OXA-232	231	JALXGM000000000
125C6	*K. pneumoniae*	2016	Rectal swab	OXA-232	231	JALXGN000000000
126I9	*K. pneumoniae*	2016	Rectal swab	OXA-232	231	JALXGO000000000
130H7	*K. pneumoniae*	2017	Rectal swab	OXA-232 + NDM-1	14	JALXGP000000000
135D8	*K. pneumoniae*	2017	Rectal swab	OXA-232	16	JALXGQ000000000
136G10	*K. pneumoniae*	2017	Rectal swab	OXA-232	231	JALXGR000000000
141A4	*K. pneumoniae*	2017	Other or unknown origin	OXA-232	231	JALXGS000000000
141F7	*K. pneumoniae*	2017	Rectal swab	OXA-232	2096	JALXGT000000000
145E7	*K. pneumoniae*	2017	Other or unknown origin	OXA-232	2096	JANJFV000000000
147E10	*K. pneumoniae*	2017	Rectal swab	OXA-232 + NDM-5	147	JALXGU000000000
154D6	*K. pneumoniae*	2017	Rectal swab	OXA-232	2096	JALXGV000000000
157H9	*K. pneumoniae*	2017	Urines	OXA-232	231	JALXGW000000000
161J6	*K. pneumoniae*	2018	Rectal swab	OXA-232 + NDM-1	11	JALXGX000000000
166D10	*K. pneumoniae*	2018	Urines	OXA-232	2096	JALXGY000000000
168E9	*K. pneumoniae*	2018	Rectal swab	OXA-232	2096	JALXGZ000000000
169A7	*K. pneumoniae*	2018	Rectal swab	OXA-232 + NDM-1	14	JALXHA000000000

Strain	Species	Year	Origin	Carbapenemase	ST	Genome Accession Number
175I6	*K. pneumoniae*	2018	Rectal swab	OXA-232	231	JALXHB000000000
181D1	*K. pneumoniae*	2018	Urines	OXA-232 + NDM-1	2497	JALXHC000000000
189D9	*K. pneumoniae*	2018	Rectal swab	OXA-232	2096	JANJFW000000000
190H10	*K. pneumoniae*	2018	Rectal swab	OXA-232	231	JALXHD000000000
200D8	*K. pneumoniae*	2019	Rectal swab	OXA-232	2096	JALXHE000000000
206G1	*K. pneumoniae*	2019	Rectal swab	OXA-232	231	JALXHF000000000
206J2	*K. pneumoniae*	2019	Rectal swab	OXA-232	2096	JALXHG000000000
208I4	*K. pneumoniae*	2019	Rectal swab	OXA-232	2096	JALXHH000000000
212D6	*K. pneumoniae*	2019	Rectal swab	OXA-232	231	JALXHI000000000
216I5	*K. pneumoniae*	2019	Rectal swab	OXA-232	2096	JALXHJ000000000
219H4	*K. pneumoniae*	2019	Urines	OXA-232 + NDM-1	2497	JALXHK000000000
225I1	*K. pneumoniae*	2019	Rectal swab	OXA-232	2096	JALXHL000000000
226C5	*K. pneumoniae*	2019	Rectal swab	OXA-232	231	JALXHM000000000
228E4	*K. pneumoniae*	2019	Rectal swab	OXA-232	2096	JALXHN000000000
229D5	*K. pneumoniae*	2019	Rectal swab	OXA-232	2096	JALXHO000000000
229I6	*K. pneumoniae*	2019	Urines	OXA-232	2096	JALXHP000000000
230D9	*K. pneumoniae*	2019	Rectal swab	OXA-232	2096	JANJFX000000000
230F5	*K. pneumoniae*	2019	Rectal swab	OXA-232	2096	JALXHQ000000000
230J4	*K. pneumoniae*	2019	Rectal swab	OXA-232	231	JALXHR000000000
233G5	*K. pneumoniae*	2019	Rectal swab	OXA-232	2096	JANJFY000000000
234H9	*K. pneumoniae*	2019	Rectal swab	OXA-232	231	JALXHS000000000
235H1	*K. pneumoniae*	2019	Urines	OXA-232	147	JALXHT000000000
236A8	*K. pneumoniae*	2019	Rectal swab	OXA-232	2096	JALXHU000000000
236D10	*K. pneumoniae*	2019	Urines	OXA-232 + NDM-5	147	JALXHV000000000
236I4	*K. pneumoniae*	2019	Urines	OXA-232 + NDM-5	147	JALXHW000000000
238E5	*K. pneumoniae*	2019	Rectal swab	OXA-232	231	JALXHX000000000
240D2	*K. pneumoniae*	2019	Rectal swab	OXA-232	2096	JALXHY000000000
244B1	*K. pneumoniae*	2020	Rectal swab	OXA-232 + NDM-5	231	JALXHZ000000000
247H10	*K. pneumoniae*	2020	Urines	OXA-232	2096	JANJFZ000000000
248B4	*K. pneumoniae*	2020	Rectal swab	OXA-232	29	JALXIA000000000
248B5	*K. pneumoniae*	2020	Rectal swab	OXA-232	29	JALXIB000000000
248F9	*K. pneumoniae*	2020	Urines	OXA-232 + NDM-1	16	JALXIC000000000
250F5	*K. pneumoniae*	2020	Rectal swab	OXA-232	273	JALXID000000000
254D2	*K. pneumoniae*	2020	Rectal swab	OXA-232 + NDM-5	231	JALXIE000000000
255E9	*K. pneumoniae*	2020	Other or unknown origin	OXA-232	231	JALXIF000000000
256B1	*K. pneumoniae*	2020	Rectal swab	OXA-232	231	JALXIG000000000
259A4	*K. pneumoniae*	2020	Urines	OXA-232 + NDM-1	2497	JALXIH000000000
263E1	*K. pneumoniae*	2020	Rectal swab	OXA-232	231	JALXII000000000
265B7	*K. pneumoniae*	2020	Rectal swab	OXA-232	2096	JALXIJ000000000
271F4	*K. pneumoniae*	2020	Other or unknown origin	OXA-232	231	JALXIK000000000
274D7	*K. pneumoniae*	2020	Rectal swab	OXA-232	2096	JALXIL000000000
276E2	*K. pneumoniae*	2021	Urines	OXA-232	231	JALXIM000000000
278E10	*K. pneumoniae*	2021	Urines	OXA-232	231	JALXIN000000000
285I1	*K. pneumoniae*	2021	Rectal swab	OXA-232 + NDM-5	147	JALXIO000000000
290D2	*K. pneumoniae*	2021	Rectal swab	OXA-232	437	JALXIP000000000
290I5	*K. pneumoniae*	2021	Rectal swab	OXA-232	New	JALXIQ000000000
292C2	*K. pneumoniae*	2021	Rectal swab	OXA-232 + NDM-1	16	JALXIR000000000

Strain	Species	Year	Origin	Carbapenemase	ST	Genome Accession Number
294H6	*K. pneumoniae*	2021	Other or unknown origin	OXA-232	15	JALXIS000000000
295I1	*K. pneumoniae*	2021	Rectal swab	OXA-232	2096	JALXIT000000000
295I3	*K. pneumoniae*	2021	Rectal swab	OXA-232	2096	JALXIU000000000
295J6	*K. pneumoniae*	2021	Rectal swab	OXA-232	395	JALXIV000000000
296D5	*K. pneumoniae*	2021	Rectal swab	OXA-232	2096	JALXIW000000000
298C6	*K. pneumoniae*	2021	Rectal swab	OXA-232	2096	JALXIX000000000
300F7	*K. pneumoniae*	2021	Rectal swab	OXA-232 + NDM-5	16	JALXIY000000000
301I6	*K. pneumoniae*	2021	Rectal swab	OXA-232 + NDM-1	16	JALXIZ000000000
303C8	*K. pneumoniae*	2021	Urines	OXA-232 + NDM-1	14	JALXJA000000000
303I7	*K. pneumoniae*	2021	Rectal swab	OXA-232	2096	JALXJB000000000
304B3	*K. pneumoniae*	2021	Rectal swab	OXA-232	512	JALXJC000000000
306A9	*K. pneumoniae*	2021	Rectal swab	OXA-232 + NDM-5	147	JALXJD000000000
307J2	*K. pneumoniae*	2021	Rectal swab	OXA-232 + NDM-5	16	JALXJE000000000
309B8	*K. pneumoniae*	2021	Rectal swab	OXA-232	2096	JALXJF000000000
311I8	*K. pneumoniae*	2021	Rectal swab	OXA-232	2096	JALXJG000000000
312C6	*K. pneumoniae*	2021	Urines	OXA-232	395	JALXJH000000000
ND	*E. coli*	2013	Rectal swab	OXA-232 + NDM-1	ND	
ND	*K. pneumoniae*	2013	Rectal swab	OXA-232 + NDM-1	ND	
ND	*K. pneumoniae*	2013	Rectal swab	OXA-232 + NDM-1	ND	
43B6	*K. pneumoniae*	2014	Rectal swab	OXA-232	ND	
44C5	*K. pneumoniae*	2014	Rectal swab	OXA-232	ND	
46A8	*E. coli*	2014	Other or unknown origin	OXA-232 + NDM-1	ND	
68B2	*E. coli*	2014	Other or unknown origin	OXA-232	ND	
111C6	*E. coli*	2016	Rectal swab	OXA-232	ND	
117C6	*E. coli*	2016	Rectal swab	OXA-232	ND	
148H7	*E. coli*	2017	Rectal swab	OXA-232 + NDM-5	ND	
227H1	*E. coli*	2019	Rectal swab	OXA-232	ND	
165B3	*C. freundii*	2018	Rectal swab	OXA-232	ND	
170F5	*C. freundii*	2018	Urines	OXA-232	ND	
188H3	*C. freundii*	2018	Urines	OXA-232	ND	
203I10	*C. freundii*	2019	Rectal swab	OXA-232	ND	
207F9	*C. freundii*	2019	Rectal swab	OXA-232 + NDM-1	ND	
213I9	*C. freundii*	2019	Rectal swab	OXA-232	ND	
229I3	*C. freundii*	2019	Rectal swab	OXA-232	ND	
230B3	*C. freundii*	2019	Rectal swab	OXA-232	ND	
236A9	*C. freundii*	2019	Rectal swab	OXA-232	ND	
263J3	*C. freundii*	2020	Rectal swab	OXA-232 + NDM-5	ND	
285D4	*C. freundii*	2021	Urines	OXA-232 + NDM-1	ND	
292G1	*C. freundii*	2021	Rectal swab	OXA-232	ND	
299H2	*C. freundii*	2021	Other or unknown origin	OXA-232	ND	
214J10	*K. aerogenes*	2019	Rectal swab	OXA-232	ND	
255E10	*K. aerogenes*	2020	Blood culture	OXA-232	ND	
85F5	*K. oxytoca*	2015	Rectal swab	OXA-232	ND	

ST, sequence type obtained by MLST; ND, not determined.

Appendix Table 2. Percentage of OXA-232 producing isolates received at the French National Reference Center for carbapenem-resistant Enterobacterales among all carbapenemase-producing Enterobacterales (CPE) or among OXA-48-like from 2013 to 2021.

Year	OXA-232 and OXA-232 +NDM (n)	% among CPE	% among OXA-48 like	OXA-232 (n)	% among CPE	% among OXA-48 like	OXA-232 + NDM (n)	% among CPE	% among OXA-48 like
2013	3	0.47%	0.59%	0	0.00%	0.00%	3	0.47%	0.59%
2014	4	0.37%	0.43%	3	0.28%	0.33%	1	0.09%	0.11%
2015	7	0.55%	0.71%	5	0.39%	0.51%	2	0.16%	0.20%
2016	15	0.97%	1.26%	14	0.90%	1.18%	1	0.06%	0.08%
2017	10	0.52%	0.69%	8	0.42%	0.56%	2	0.10%	0.14%
2018	10	0.37%	0.52%	8	0.30%	0.42%	2	0.07%	0.10%
2019	30	0.99%	1.40%	26	0.86%	1.22%	4	0.13%	0.19%
2020	16	0.73%	1.14%	12	0.54%	0.86%	4	0.18%	0.29%
2021	23	0.94%	1.40%	17	0.69%	1.03%	6	0.25%	0.36%
2013–2018	49	0.54%	0.70%	38	0.42%	0.55%	11	0.12%	0.16%
2019–2021	69	0.91%	1.33%	55	0.72%	1.06%	14	0.18%	0.27%

Appendix Figure. Global characterization (sequence type, year of isolation, β-lactamase content) of nonduplicate 95 OXA-232–producing *Klebsiella pneumoniae* analyzed at the National Reference Center for Carbapenem-Resistant Enterobacterales, France, 2013–2021. Scale bar indicates the number of SNP per position of common sequences. CMY-6, variant of *C. freundii* intrinsic cephalosporinase; CTX-M, cefotaximase–Munich extended-spectrum β-lactamase; OXA, oxacillinase; NDM, New Delhi metallo-β-lactamase; ST, sequence type, TEM, Temoniera β-lactamase.
